# Evaluation of an improved tool for non-invasive prediction of neonatal respiratory morbidity based on fully automated fetal lung ultrasound analysis

**DOI:** 10.1038/s41598-019-38576-w

**Published:** 2019-02-13

**Authors:** Xavier P. Burgos-Artizzu, Álvaro Perez-Moreno, David Coronado-Gutierrez, Eduard Gratacos, Montse Palacio

**Affiliations:** 1 BCNatal - Barcelona Center for Maternal-Fetal and Neonatal Medicine (Hospital Clínic and Hospital Sant Joan de Deu, University of Barcelona), Barcelona, Spain; 2 Transmural Biotech S. L. Barcelona, Barcelona, Spain; 3 Institut D’Investigacions Biomèdiques August Pi i Sunyer, IDIBAPS, Spain; 4 Center for Biomedical Research on Rare Diseases (CIBER-ER), Barcelona, Spain

## Abstract

The objective of this study was to evaluate the performance of a new version of quantusFLM®, a software tool for prediction of neonatal respiratory morbidity (NRM) by ultrasound, which incorporates a fully automated fetal lung delineation based on Deep Learning techniques. A set of 790 fetal lung ultrasound images obtained at 24 + 0–38 + 6 weeks’ gestation was evaluated. Perinatal outcomes and the occurrence of NRM were recorded. quantusFLM® version 3.0 was applied to all images to automatically delineate the fetal lung and predict NRM risk. The test was compared with the same technology but using a manual delineation of the fetal lung, and with a scenario where only gestational age was available. The software predicted NRM with a sensitivity, specificity, and positive and negative predictive value of 71.0%, 94.7%, 67.9%, and 95.4%, respectively, with an accuracy of 91.5%. The accuracy for predicting NRM obtained with the same texture analysis but using a manual delineation of the lung was 90.3%, and using only gestational age was 75.6%. To sum up, automated and non-invasive software predicted NRM with a performance similar to that reported for tests based on amniotic fluid analysis and much greater than that of gestational age alone.

## Introduction

Neonatal Respiratory Morbidity (NRM) is the leading cause of mortality and morbidity associated with prematurity^1–3^. NRM can be assessed through Fetal Lung Maturity (FLM) estimation, helping to decide on the use of corticosteroids or plan place and time of elective delivery in late pregnancy complications^4–8^. Traditional clinical options for FLM estimation are either to use gestational age directly as a proxy FLM estimator or studying several components of the amniotic fluid^9,10^ through amniocentesis.

For decades, several approaches were attempted to estimate FLM non-invasively, involving direct image gray scale measurements^11,12^, lung tissue motion^13,14^ or the relation between lung and liver tissues,^15^ but none of these studies showed sufficient diagnostic accuracy for a real clinical use. More recently, quantitative texture analysis, a powerful technique to extract information from medical images and quantify tissue changes, was applied to the prediction of FLM^16–18^. Based on these principles, a non-invasive FLM estimator software (quantusFLM®, Transmural Biotech, Barcelona, Spain) was developed. This software has proven to have prediction accuracies similar to that of amniocentesis, first in single-center studies^19,20^ and recently in a large multi-center study with more than 700 deliveries^21^. Shortly after these first studies were published, the original technology behind quantusFLM® was improved by incorporating Deep Learning techniques^22^, which have revolutionized image processing in the last few years. This has resulted in a novel algorithm (i.e. version 3.0), designed to improve the prediction of NRM and to allow automatic identification and segmentation of the fetal lung, thereby avoiding the need for manual delineation as required in earlier versions of the software.

In this study, we evaluated the performance of this new algorithm to predict NRM in a cohort of 790 fetuses where lung images were obtained within 48 hours of delivery and perinatal outcomes were recorded, at a gestational age range of 24 + 0–38 + 6 weeks’ gestation. In addition, we evaluated in the same cohort the predictive value of the same texture analysis algorithm but using a manual delineation of the fetal lung, and predictive performance of gestational age alone.

## Materials and Methods

### Patient recruitment and image acquisition protocol

A cohort of images (N = 730) from a previous multi-center study^21^ containing cases from 20 centers worldwide was used^21^. This set was further augmented with additional cases (N = 60) recruited at BCNatal (Hospital Clinic and Hospital Sant Joan de Deu, Barcelona) under the same protocol (2011/6291, 2013/8892) as the previous study.

Patients included in the study were receiving care in the participating institutions and enrolled either in a specific protocol for the evaluation of fetal lung maturity, in studies involving the use of fetal ultrasound or in studies where ultrasound was used as part of the clinical management approved by the local review boards. All patients included in the study gave written informed consent for the use of ultrasound images and perinatal data. All the methods hereby explained were performed in accordance with the relevant guidelines and regulations and approved, together with the study protocol, by the coordinator’s Institutional Review Board (Comité de ética de investigación clínica CEIC 2011/6291, 2013/8892).

A detailed description of the image acquisition protocol and definitions used of clinical outcomes is fully described in a previous study^21^. Briefly, eligible cases included pregnancies between 24 + 0 and 38 + 6 weeks of gestation in which an ultrasound was obtained within 48 h of delivery. Cases were considered non-eligible if corticosteroids were used for lung maturity between the image acquisition and delivery, when maternal BMI was ≥35 and when fetuses had known congenital malformations. Neonates with conditions that could directly predispose or lead to NRM irrespective of lung maturity were also excluded.

**Figure 1:**
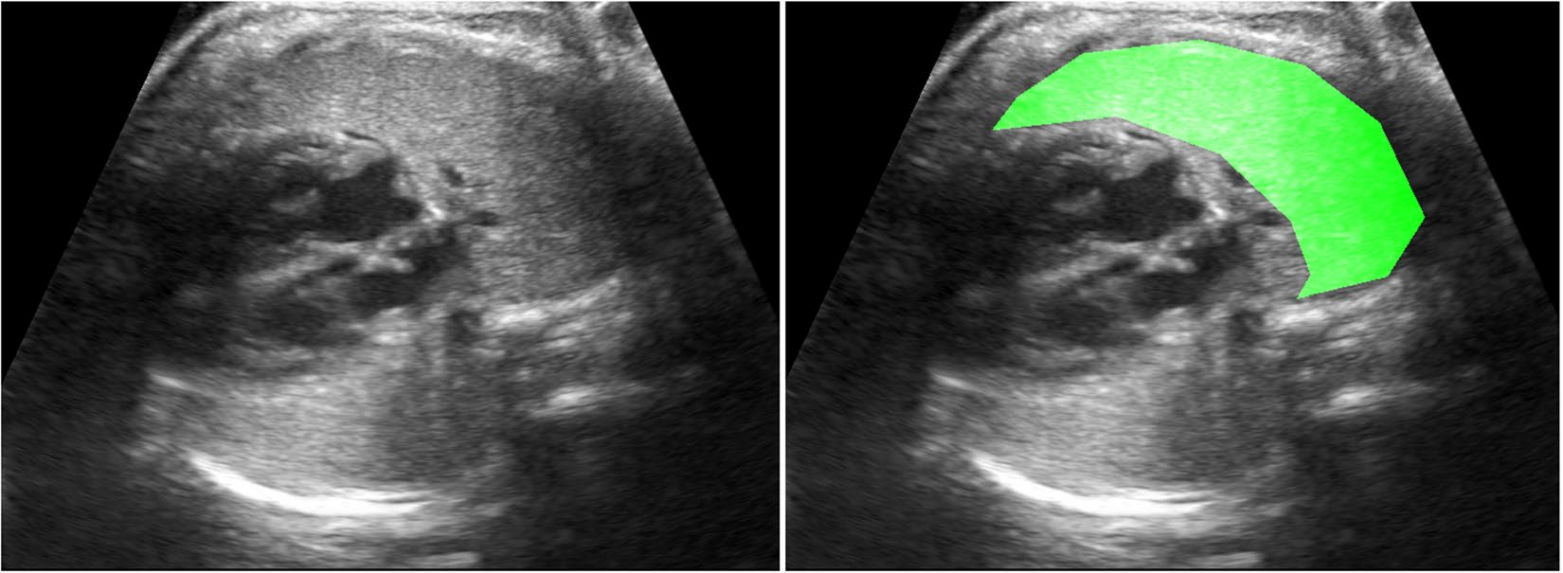
Example fetal lung ultrasound image and ROI marking the entire proximal lung.

Ultrasound images were obtained following a detailed acquisition protocol: an axial section of the fetal thorax at the level of the four-chamber cardiac view was magnified by adjusting only depth, but not the zoom option, until the thorax occupied about two thirds of the screen, avoiding obvious acoustic shadows from the fetal ribs (Fig. 1). The use of tissue harmonic imaging and adjustment of image settings such as gain, frequency and gain compensation were left to the discretion of the physician performing the ultrasound scan. All study images were inspected for image quality control and stored in the original Digital Imaging and Communication in Medicine (DICOM) format.

The primary clinical outcome of the study was NRM, including respiratory distress syndrome (RDS) or transient tachypnea of the newborn (TTN). Respiratory distress syndrome was defined based on clinical criteria including grunting, nasal flaring, tachypnea, chest wall retraction, and the need for supplemental oxygen together with typical chest radiography findings and admission to the neonatal intensive care unit for respiratory support^2^. TTN was diagnosed based on early respiratory distress (isolated tachypnea, rare grunting, minimal retraction) and a chest X-ray showing hyper-aeration of the lungs and prominent pulmonary vascular patterns^23^.

Gestational age was calculated for each patient based on the crown-rump length at first trimester ultrasound.

### Image processing

DICOM images were processed using the new quantusFLM®, which automatically delineated a region of interest (ROI) in the fetal lung and calculated a NRM risk score. The same set of 790 images was then analyzed using the same texture analysis algorithm as above but from a manual delineation of the ROI instead. The manual ROIs were delineated by an expert, using a Graphical User Interface (GUI) developed in MATLAB. An example of the ROIs used is shown in Fig. 1.

### Statistical analysis

Characteristics of the study population were described using mean and standard deviation or number and percentage where appropriate. Missing information on main general variables such as race, baby gender, delivery mode, etc. were first tested using Missing Completely At Random (MCAR) via Little’s test^24^, and then blanks were filled using multiple imputation.

#### Automatic vs manual ROI delineation accuracy and reproducibility

In order to evaluate the accuracy of automatic delineation of the fetal lung, automatically and manually delineated ROIs were compared using the most typical segmentation metric: pixel-to-pixel mean intersection over union. This metric measures the number of overlapping pixels between two ROIs as a percentage between 0 and 100, where 50% is usually considered the minimum satisfactory overlap percentage to call two ROIs “similar”. Furthermore, we evaluated the intra-observer reproducibility of manually delineated ROIs in a random sample of 100 images. Images were delineated twice by the same expert user and the area overlap was measured.

#### NRM prediction performance

To evaluate NRM prediction, output continuous NRM risk scores were binarized using the optimal cut-off point threshold, resulting in a dichotomic “high” or “low” NRM prediction. Optimal cut-off threshold was computed as that maximizing F1-Score using the entire dataset. F1-Score is an accuracy metric which measures the harmonic average between Sensitivity and Positive Predictive Value and is defined as (2*TP)/(2*TP + FP + FN). When prevalence is far from 50% as in NRM, F1-Score should be the preferred metric for measurement of overall performance^25^: instead of focusing equally on negatives and positives as standard Accuracy does, it balances Sensitivity and Positive Predictive Value to better judge the real usefulness of the prediction. From binarized NRM prediction, all classical performance indexes (Sensitivity, Specificity, etc.) were calculated at three different groups based on gestational age ([25.0–33.6], [34.0–38.6] and [34.0–36.6]).

#### Comparison of the performance among different methods

We compared the performance on NRM prediction of quantusFLM® (with automatic fetal lung delineation) against both if manual ROIs were used as input to quantusFLM® instead and against a scenario where only gestational age was available. For fairness of comparison, optimal cut-off thresholds were computed independently in each case as those maximizing F1-Score using the entire dataset, as before. Apart from comparing NRM prediction performance, we performed McNemar’s Test^26^ between the full proposed system and the other options (manual ROIs and gestational age alone), to establish quantitatively the statistical differences between the output predictions. Finally, for completeness, to establish if there was a relationship between performance and image quality, we evaluated performance on a subset of the images that were subjectively qualified as having ‘optimal image quality’ by the research team.

## Results

### Final dataset composition

Among the 790 pregnancies, there were 107 cases of NRM (13.5%) and 683 controls. Table 1 shows the general clinical features of the study groups (see Supporting Information Tables S1 -general characteristics-, S2 – perinatal and neonatal outcomes- and S3 – respiratory support and morbidity- for more in-depth analysis).

**Table 1:** General characteristics of the study population.

	All (n = 790)	GA at scan		
		24.0–33.6 (n = 174)	34.0–36.6 (n = 197)	34.0–38.6 (n = 616)
Maternal Age	31.7 (5.7)	31.7 (5.5)	31.4 (5.9)	31.7 (5.7)
Nulliparity	416 (52.7%)	88 (50.5%)	108 (54.8%)	328 (53.2%)
Multiple pregnancy	75 (8.9%)	25 (14.5%)	13 (6.5%)	50 (8.1%)
Maternal or fetal relevant conditions				
Preterm labor	49 (6.2%)	27 (15.5%)	18 (9.2%)	22 (3.5%)
PPROM	158 (20%)	76 (43.7%)	64 (32.5%)	82 (13.3%)
preeclampsia	124 (15.7%)	41 (23.6%)	39 (19.8%)	83 (13.5%)
IUGR	148 (18.7%)	35 (20.1%)	35 (17.8%)	113 (18.3%)
Gestational Age at delivery (weeks)	36.0 (2.6)	31.4 (2.2)	35.5 (0.7)	37.2 (1.2)
Mode of delivery				
Spontaneous vaginal delivery	315 (39.9%)	54 (31.0%)	88 (44.7%)	261 (42.4%)
Elective cesarean section	279 (35.3%)	75 (43.1%)	61 (31.0%)	204 (33.1%)
Birthweight (g)	2517 (755)	1554 (483)	2368 (445)	2787 (576)
Corticosteroid administered	225 (28%)	146 (84%)	59 (30%)	79 (12.8%)
Lapse between last steroid dose and scan (days)	9.9 (15.4)	6.2 (11.4)	12.6 (17.4)	16.6 (19.2)
NICU admission	247 (31.2%)	152 (87.4%)	71 (36.0%)	95 (15.4%)
Neonatal Respiratory Morbidity	107 (13.5%)	72 (41.3%)	31 (15.7%)	35 (5.6%)

Mean (SD) or n (%) when appropriate. PPROM: preterm premature rupture of membranes. IUGR: intrauterine growth restriction. NICU: neonatal intensive care unit.GA, gestational age.

### Automatic versus manual ROI delineation accuracy and reproducibility

**Figure 2:**
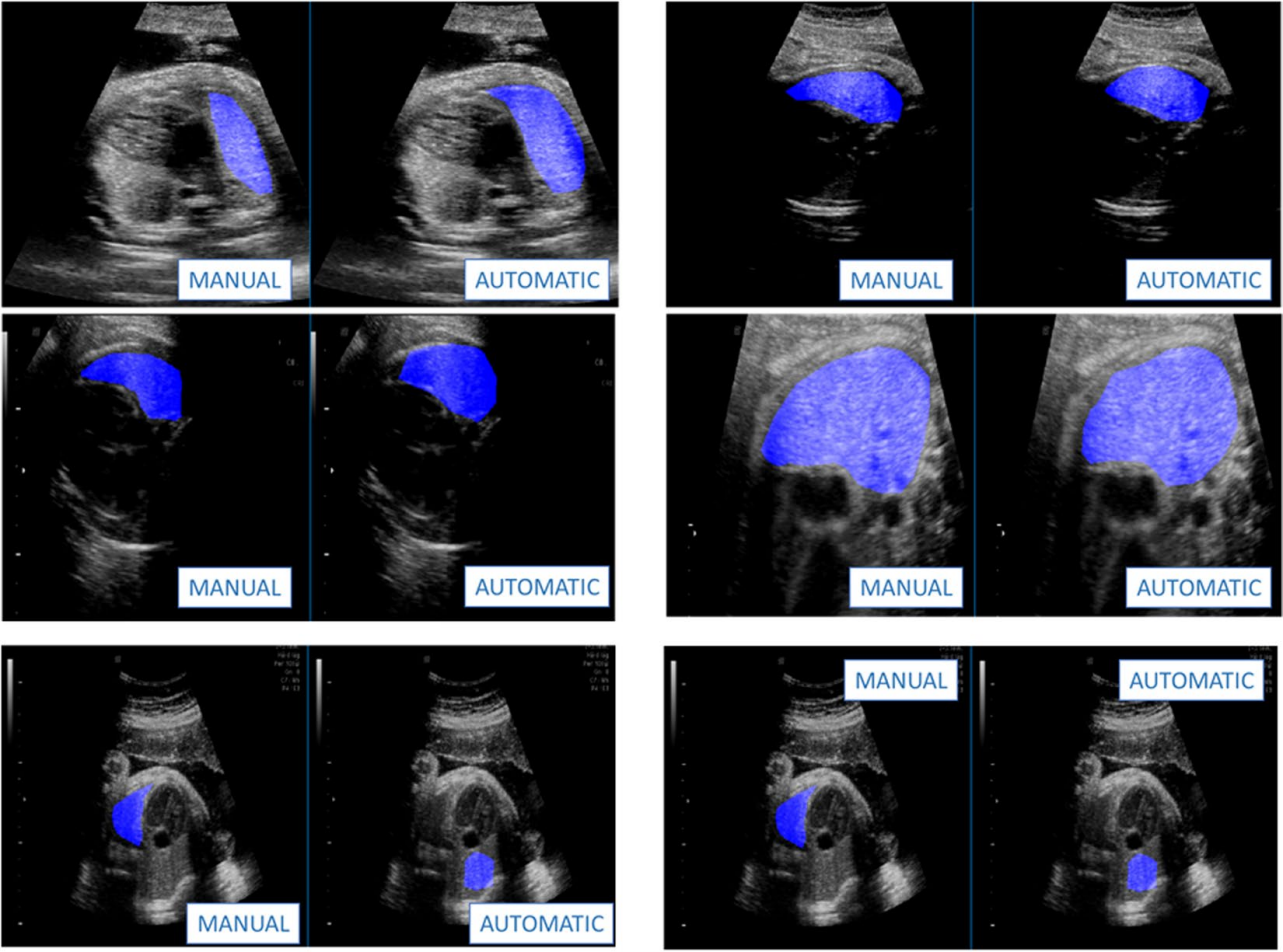
Example automatic ROI segmentation of test images. Top 2 rows: regular success cases. Bottom row: example “failure” cases. The automatic segmentation extracts the fetal lung correctly in all test images and ensures 100% reproducibility of results given same image. Even when it disagrees with human ROIs it is delineating fetal lung and not another organ.

Table 2 shows the accuracy and reproducibility of quantusFLM®’s automatic ROI delineation. Fig. 2 shows some visual examples comparing the automatic and manual ROIs. The automatic delineation reached 93% average overlap with expert’s manual ROIs, with only 1.5% of the ROIs falling below 50% overlap (12/790). In terms of reproducibility, while the automatic delineation ensures 100% (the ROI is always the same given the same image), experts changed on average 12% of the ROI pixels (reproducibility of 88%) on the subset of 100 images they delineated twice.

**Table 2:** Automatic vs manual fetal lung ROI delineation accuracy and reproducibility.

Automatic delineation ACCURACY (compared with expert’ ROIs)		
Overlap average	93% (std = 4.5%)	
Number of Images with overlap <50%	12 (1.5%)	
**REPRODUCIBILITY (comparison with itself)**		
	**AUTOMATIC**	**MANUAL**
Overlap average	100% (0%)	88% (std = 2.0%)
Number of Images with overlap <50%	0 (0%)	0 (0%)

Mean (SD) or n (%) where appropriate.

### NRM prediction performance

Table 3 shows the performance of the three approaches used. Using F1-Score as main reference, texture analysis on automatically and manually delineated ROIs resulted in scores of 69.4% and 65.5% respectively. GA alone (optimal cut-off point at GA <= 35.5) resulted in a score of 49.6%. McNemar’s Test result between manual and automatic NRM predictions was p = 0.07, indicating some similarity. McNemar’s Test between texture analysis by either method and GA alone was p < 0.01, indicating a statistical difference between them.

**Table 3:** Performance on NRM prediction.

	quantusFLM®				quantusFLM® from manual ROIs				Using GA only			
	All	24.0–33.6	34.0–36.6	34.0–38.6	All	24.0–33.6	34.0–36.6	34.0–38.6	All	24.0–33.6	34.0–36.6	34.0–38.6
N	790	174	197	616	790	174	197	616	790	174	197	616
NRM	107 (13.5%)	72 (41.4%)	31 (15.7%)	35 (5.7%)	107 (13.5%)	72 (41.4%)	31 (15.7%)	35 (5.7%)	107 (13.5%)	72 (41.4%)	31 (15.7%)	35 (5.7%)
SENS	71.0% (76/107)	75.0% (54/72)	64.5% (20/31)	62.9% (22/35)	68.2% (73/107)	76.4% (55/72)	51.6% (16/31)	51.4% (18/35)	88.8% (95/107)	100.0% (72/72)	74.2% (23/31)	65.7% (23/35)
SPEC	94.7% (647/683)	87.3% (89/102)	88.6% (147/166)	96.0% (558/581)	93.7% (640/683)	86.3% (88/102)	84.9% (141/166)	95.0% (552/581)	73.5% (502/683)	0.0% (0/102)	52.4% (87/166)	86.4% (502/581)
PPV	67.9% (76/112)	80.6% (54/67)	51.3% (20/39)	48.9% (22/45)	62.9% (73/116)	79.7% (55/69)	39.0% (16/41)	38.3% (18/47)	34.4% (95/276)	41.4% (72/174)	22.5% (23/102)	22.5% (23/102)
NPV	95.4% (647/678)	83.2% (89/107)	93.0% (147/158)	97.7% (558/571)	95.0% (640/674)	83.8% (88/105)	90.4% (141/156)	97.0% (552/569)	97.7% (502/514)	0.0% (0/0)	91.6% (87/95)	97.7% (502/514)
ACC	91.5% (723/790)	82.2% (143/174)	84.8% (167/197)	94.2% (580/616)	90.3% (713/790)	82.2% (143/174)	79.7% (157/197)	92.5% (570/616)	75.6% (597/790)	41.4% (72/174)	55.8% (110/197)	85.2% (525/616)
F1- Score	69.4% (152/219)	77.7% (108/139)	57.1% (40/70)	55.0% (44/80)	65.5% (146/223)	78.0% (110/141)	44.4% (32/72)	43.9% (36/82)	49.6% (190/383)	58.5% (144/246)	34.6% (46/133)	33.6% (46/137)
LR+	13.5	5.9	5.6	15.9	10.8	5.6	3.4	10.3	3.4	1.0	1.6	4.8
LR-	0.3	0.3	0.4	0.4	0.3	0.3	0.6	0.5	0.2	0.0	0.5	0.4

NRM = Neonatal Respiratory Morbidity; SENS = Sensitivity, SPEC = Specificity;PPV = Positive Predictive Value; NPV = Negative Predictive Value; ACC = Accuracy; LR+ = Positive Likelihood Ratio; LR- = Negative Likelihood Ratio.

F-1 score in the subset of 372 images (47% of the data) which were considered as having “optimal image quality” by the research team was 71.3%, only 1.9% higher than on the full set.

## Discussion

In this study we evaluated an improved version of existing commercial software for NRM prediction, which now includes an automatic delineation of the fetal lung and novel Deep Learning ultrasound image processing techniques. The results showed that automatic delineation of the fetal lung was as reliable as manual delineation, with the advantage of improved repeatability. The predictive performance of the software achieved results that improved those reported for previous versions of the same technology. Finally, the results illustrate that a computer assisted method improves significantly the prediction of NRM as based merely on gestational age.

Results of this study suggest that the automated delineation method achieved similar or slightly better prediction rates in relation with the reported performance in a very similar population for the previous version of quantusFLM®^21^. In the overall population evaluated, results showed improvements by about 5% in accuracy and 9% in F1-score, and in the 34.0–38.6 age range, accuracy was higher by 5% and F-1 score was higher by 17% in comparison with previously reported data. Moreover, although a direct comparison was not performed, the data again points towards similar or better prediction performance of quantusFLM® as compared with laboratory methods in amniotic fluid^27–32^, see Supporting Information Table S4. The best performing method, Lecithin/sphingomyelin ratio, has a reported accuracy 10% lower (81.6% compared to 91.7%) and a 22.6% lower F1-Score (46.8% compared to 69.4%). In addition, direct comparison with the use of gestational age alone as a predictor of the risk of NRM showed a remarkable improvement. Accuracy was improved by 16% and F-1 score by 19.8%. Finally, the similar performance observed in optimal and suboptimal quality images suggests that the system is robust to perform well in real conditions.

The software evaluated in this study introduced a fully-automated delineation of the fetal lung, thereby avoiding the need for manual delineation as required in earlier versions of the software^19–21^. The comparison between manual and automatic segmentation demonstrated that automatic delineation did not reduce the predictive performance of the software. Automatic medical ultrasound image segmentation has been widely studied^33,34^, with notable success examples such as prostate^35^ or breast cancer images^36^. Segmentation of fetal ultrasound images has also been studied, mostly as a tool for the automatic evaluation of fetal biometries, estimating structures such as abdomen, head, femur or the whole fetus^37,38^. Yet, as far as we know, this is the first study reporting reliable fetal lung segmentation from ultrasound images.

From a clinical perspective, the use of a noninvasive test for FLM can be particularly relevant in late preterm deliveries, which represent 5–10% of pregnancies in most healthcare systems. As far as around 23% of cases of late preterm deliveries did not have a clear indication or were delivered after a non-evidence based indication^39,40^. Reliable information about the risks of NRM might be crucial to plan the place and timing of delivery. Furthermore, this information would assist in the decision of using antenatal corticosteroids. As recently shown in a randomized trial, antenatal corticosteroids reduced by about 20% the risk of NRM in late-preterm deliveries^8^. Considering the prevalence of late-preterm delivery, this small benefit might represent thousands of cases yearly and a remarkable fraction of neonatal health-care costs. Thus, a strong reason against the generalized use of corticosteroids in late-preterm pregnancies is that a modest benefit should be weighed against the potentially important risks of corticosteroids in neurodevelopment and fetal metabolic programming^41–43^. Therefore, efforts have to be raised not to overuse them in cases which do not meet the strict criteria of the study published by Gyamfi-Bannerman *et al*.^8,44^. Furthermore, the balance between benefits and risks has to be evaluated when repeated doses long after an initial dose are considered or if an early term elective cesarean delivery is planned^45^. A noninvasive technique for predicting FLM might select patients eligible for the administration of corticosteroids late in pregnancy. In addition, a non-invasive technique would avoid the fear and discomfort of amniocentesis, which has been another reason commonly given for not evaluating FLM by classical methods in amniotic fluid. Thus, a non-invasive tool to determine the individual risk of each baby to develop NRM would allow a selective use of corticosteroids in this context. Otherwise, with a systematic administration policy, almost 90% of late-preterm deliveries would receive corticosteroids unnecessarily. On another hand, a remarkable fraction of newborns will effectively suffer from NRM despite corticosteroid administration. In this study for example, corticosteroids were administered to 71% of fetuses that ended up having NRM anyway (76/107). Predicting this risk might allow a better planning of strategies of neonatal support. Thus, the use of a non-invasive tool that individualizes the risk of NRM would allow selecting cases for corticosteroid administration, while identifying a high-risk group that could develop NRM despite the use of corticosteroids. Finally, it would be interesting to study if this tool can be used to detect changes in the fetal lung after steroid administration. However, we believe this is something that needs to be addressed appropriately in a separate, well focused study. Since it can be assumed that changes in FLM occur progressively after steroid administration, it is plausible to think that changes in texture parameters may not be detectable until a particular threshold and we currently do not know how close to the steroids administration these changes might be detected. This would need to be studied.

This study has strengths and limitations. Images were collected at multiple centers around the world using different machines and leaving configuration in the hands of each technician, therefore mimicking real clinical conditions. The final dataset statistics are consistent with those of the previous study^21^ but with the addition of 60 patients, 50 of which were late pre-term and early-term, which allowed to better assess the performance of the software in this specific group. In this study we addressed a common criticism that systems for predicting NRM should be compared with the performance of gestational age alone. Among the limitations of this study, the rate of NRM in the preterm group was around 15% which is a higher rate compared to the one described in other studies^2^. In addition, we acknowledge that although the results suggest a clear improvement in relation with the published performance of methods based on amniotic fluid analysis, a direct comparison on the same patients was not undertaken. Finally, the method tested in this study uses an indirect approach to estimate lung maturity. By definition, prenatal prediction of NRM is hampered by the fact that the outcome is largely, but not exclusively, determined by the fetal lung maturity status. Thus, in circumstances such as neonatal sepsis, malformations potentially affecting lung function or intrapartum hypoxic-ischemic events, newborns with normal lung maturity in utero may present respiratory impairment. Also, specific conditions such as fetal growth restriction, multiple pregnancy, diabetes or premature rupture of membranes were not analyzed separately. Differences in the performance of quantusFLM® in these subgroups cannot be excluded and requires further research.

To conclude, a software system incorporating Deep Learning techniques improved the prediction of NRM and allowed automatic identification and segmentation of the fetal lung, thereby simplifying clinical use. Noninvasive assessment of FLM would allow selecting patients at high risk of NRM among late preterm deliveries, in order to maximize benefits and minimize the risks of antenatal corticosteroids administration and to improve planning of the place and timing of delivery. While the findings here reported confirm and expand previous studies and strongly support the use of noninvasive techniques for the prediction of NRM in the clinical setting, further studies confirming these results are strongly required.

## Supplementary information

Supplementary Info

## Data Availability

The dataset generated and analysed during the current study is not publicly available due to restrictions according to patient privacy regulations, but are available from the corresponding author on reasonable request.
