# Supplementary material for: Evaluation of an improved tool for non-invasive prediction of neonatal respiratory morbidity based on fully automated fetal lung ultrasound analysis
Source: Sci Rep. 2019 Feb 13;9:1950. doi: 10.1038/s41598-019-38576-w (PMC6374419; doi:10.1038/s41598-019-38576-w)
Supplement: Supplementary file 1 — Supplementary Info [file 41598_2019_38576_MOESM1_ESM.docx]

Evaluation of an improved tool for non-invasive prediction of neonatal respiratory morbidity based on fully automated fetal lung ultrasound analysis: Supporting information.

Xavier P. Burgos-Artizzu, Álvaro Perez-Moreno, David Coronado-Gutiérrez,

Eduard Gratacós, Montse Palacio.

**S1 Table. Demographic and pregnancy characteristics of the study population.**

Mean (SD) or n(%) when appropriate. *Hypothyrodism, hypertensive disorders,

Placenta previa, Lupus, Human immunodeficiency virus positive, assessment of fetal

wellbeing, fetal presentation. PPROM: preterm premature rupture of membranes.

IUGR: intrauterine growth restriction. GA, gestational age;

|  |  | GA at scan | | |
| --- | --- | --- | --- | --- |
|  | All (n=790) | 24.0-33.6 (n=174) | 34.0-36.6 (n=197) | 34.0-38.6 (n=616) |
| Maternal Age | 31.7 (5.7) | 31.7 (5.5) | 31.4 (5.9) | 31.7 (5.7) |
| Nulliparity | 416 (52.7%) | 88 (50.5%) | 108 (54.8%) | 328 (53.2%) |
| Ethnicity |  |  |  |  |
| Caucasian | 538 (68.1%) | 126 (72.4%) | 146 (74.1%) | 412 (66.9%) |
| Asian | 45 (5.6%) | 1 (0.5%) | 8 (4.0%) | 44 (7.1%) |
| African | 44 (5.6%) | 10 (5.7%) | 13 (6.6%) | 34 (5.5%) |
| Hispanic | 125 (15.8%) | 27 (15.5%) | 25 (12.7%) | 98 (15.9%) |
| Other | 38 (4.8%) | 9 (5.2%) | 6 (3.0%) | 29 (4.7%) |
| Multiple pregnancy | 75 (8.9%) | 25 (14.5%) | 13 (6.5%) | 50 (8.1%) |
| Maternal or fetal relevant conditions |  |  |  |  |
| Preterm labor | 49 (6.2%) | 27 (15.5%) | 18 (9.2%) | 22 (3.5%) |
| PPROM | 158 (20%) | 76 (43.7%) | 64 (32.5%) | 82 (13.3%) |
| preeclampsia | 124 (15.7%) | 41 (23.6%) | 39 (19.8%) | 83 (13.5%) |
| IUGR | 148 (18.7%) | 35 (20.1%) | 35 (17.8%) | 113 (18.3%) |
| Pre-gestational diabetes | 17 (2.1%) | 2 (1.1%) | 5(2.5%) | 15 (2.4%) |
| Antepartum hemorrhage | 10 (1.2%) | 3 (1.7%) | 1 (0.9%) | 7 (1.1%) |
| Other* | 164 (20.7%) | 32 (18.4%) | 40 (20.3%) | 132 (21.4%) |

**S2 Table. Perinatal and neonatal outcomes of the newborns included in the study.** Mean (SD) or n(%) when appropriate. NICU: neonatal intensive care unit.

|  |  | GA at scan | | |
| --- | --- | --- | --- | --- |
|  | All (n=790) | 24.0-33.6 (n=174) | 34.0-36.6 (n=197) | 34.0-38.6 (n=616) |
| Gestational Age at delivery (weeks) | 36.0 (2.6) | 31.4 (2.2) | 35.5 (0.7) | 37.2 (1.2) |
| Ultrasound-to-delivery lapse of time (days) | 0.6 (0.7) | 0.8 (0.7) | 0.6 (0.7) | 0.6 (0.6) |
| Mode of delivery |  |  |  |  |
| Spontaneous vaginal delivery | 315 (39.9%) | 54 (31.0%) | 88 (44.7%) | 261 (42.4%) |
| Operative vaginal delivery | 50 (6.3%) | 4 (2.3%) | 16 (8.1%) | 46 (7.4%) |
| Non-elective cesarean section | 146 (18.5%) | 40 (23.0%) | 33 (16.7%) | 106 (17.2%) |
| Elective casarean section | 279 (35.3%) | 75 (43.1%) | 61 (31.0%) | 204 (33.1%) |
| Birthweight (g) | 2517 (755) | 1554 (483) | 2368 (445) | 2787 (576) |
| Female gender | 395 (50%) | 74 (42.5%) | 107 (54.3%) | 321 (52.1%) |
| Apgar at 5min < 7 | 10 (1.2%) | 7 (4%) | 1 (0.5%) | 3 (0.5%) |
| pH UA 7.00-< 7.10 | 20 (2.5%) | 7 (4.0%) | 7 (3.6%) | 13 (2.1%) |
| Hyperbilirrubinemia (phototherapy) | 155 (19.6%) | 80 (46.0%) | 47 (23.8%) | 75 (12.1%) |
| Other relevant conditions: |  |  |  |  |
| Apnea | 20 (2.5%) | 20 (11.4%) | 0 (0%) | 0 (0%) |
| Bronchopulmonary displasia | 9 (1.1%) | 8 (4.6%) | 1 (0.5%) | 1 (0.1%) |
| Persistent Pulmonary hypertension | 3 (0.4%) | 2 (1.1%) | 1 (0.5%) | 0 (0%) |
| Intraventricular hemorrhage (III or IV) | 5 (0.6%) | 4 (2.3%) | 0 (0%) | 1 (0.1%) |
| Necrotizing enterocolitis | 3 (0.4%) | 3 (1.7%) | 0 (0%) | 0 (0%) |
| Neonatal death < 28 days | 3 (0.4%) | 3 (1.7%) | 0 (0%) | 0 (0%) |
| NICU admission | 247 (31.2%) | 152 (87.4%) | 71 (36.0%) | 95 (15.4%) |
| Length of stay at NICU | 13.9 (21.9) | 19.0 (26.7) | 6.4 (3.7) | 5.8 (3.5) |
| Discharged alive from NICU | 244/247 (98.7%) | 149/152 (98.0%) | 57/57 (100%) | 95/95 (100%) |

**S3 Table. Characteristics of the respiratory support and respiratory morbidity.** Mean (SD) or n(%) when appropriate. CPAP: continuous positive airway pressure.

NIV/BPAP: non-invasive ventilation/Bi-level positive airway pressure.

|  |  | GA at scan | | |
| --- | --- | --- | --- | --- |
|  | All (n=790) | 24.0-33.6 (n=174) | 34.0-36.6 (n=197) | 34.0-38.6 (n=616) |
| Need for respiratory support (any) | 121  (15.3%) | 87  (50%) | 26  (13.2%) | 34  (5.5%) |
| Oxygen therapy ≥ 40% | 58 (7.3%) | 38 (21.8%) | 13 (6.6%) | 20 (3.2%) |
| CPAP | 122 (15.4%) | 92 (52.9%) | 26 (13.2%) | 30 (4.9%) |
| NIV/BPAP | 23 (2.9%) | 22 (12.6%) | 0 (0%) | 1 (0.1%) |
| Intubation required | 33 (4.2%) | 27 (15.5%) | 4 (2.0%) | 6 (0.9%) |
| Days of intubation (if any) | 4.8 (7.5) | 5.6 (8.3) | 1 (0) | 1.86 (1.3) |
| HFV (high frequency ventilation) | 12 (1.5%) | 10(5.7%) | 2 (1.0%) | 2 (0.3%) |
| Surfactant use | 35(4.4%) | 32 (18.4%) | 2 (1.0%) | 3 (0.5%) |
| Doses of surfactant (if any) | 1.46 (0.7) | 1.44 (0.7) | 2 (1) | 1.67 (0.9) |
| Neonatal Respiratory Morbidity | 107 (13.5%) | 72(41.3%) | 31 (15.7%) | 35 (5.6%) |

**S4 Table. Summary of performance of invasive tests in amniotic fluid used to predict neonatal respiratory morbidity (data from ^23^) and their comparison with the reported quantusFLM results of this study.**

|  | ACC | SENS | SPEC | PPV | NPV | F1-Score |
| --- | --- | --- | --- | --- | --- | --- |
| L/S | 81.6% | 74.6% | 82.5% | 34.1% | 96.4% | 46.8% |
| PG | 57.5% | 82.7% | 54.4% | 18.0% | 96.3% | 29.6% |
| LBC | 75.4% | 84.2% | 74.4% | 27.9% | 97.6% | 41.9% |
| TDxII | 78.7% | 88.5% | 77.7% | 28.5% | 98.5% | 43.1% |
|  |  |  |  |  |  |  |
| quantusFLM | 91.5% | 71.0% | 94.7% | 67.9% | 95.4% | 69.4% |

L/S: lecithin/sphingomyelin ratio; PG: phosphatidylglycerol; LBC: lamellar body count; TDxII:surfactant/albumin ratio.
